# Elements of Pathology and Therapeutics; Being the Outlines of a Work Intended to Ascertain the Nature, Causes, and Most Efficacious Modes of Prevention and Cure of the Greater Number of the Diseases Incidental to the Human Frame; Illustrated by Numerous Cases and Dissections

**Published:** 1816-01

**Authors:** 


*Elements of Pathology and Therapeutics; being the Outlines of a Work intended to ascertain the Nature, Causes, and most efficacious Modes of Prevention and Cure of the greater Number of the Diseases incidental to the Human Frame; illustrated by numerous Cases and Dissections.* By CALEB HILLIER PARRY, M. D. F. R. S. Member of the College of Physicians of London; Physician to the General Hospital at Bath, &c. &c. Vol I. General Pathology. 8vo. pp. 464. London, 1815.

IT gives us unusual pleasure to have an opportunity of introducing, through the First Number of the "Medico-Chirurgical Review," a work which we confidently predict will stand a monument of fame to the author, "Ære perennius," and an honour to the age and country which gave it birth! We are not wont to be lavish of our encomiums where they are not justly merited; and as we cannot yet have learned any thing of public sentiment respecting a publication just issued from the press, we hereby stake our critical judgement and penetration, that whatever cavils and objections may be started against certain doctrines and opinions of our author, the work itself will defy the ordeal of criticism, and finally receive from the profession at large, a sentence the most honorable and gratifying that an author can wish, or the public voice confer. It is a work, indeed, which may justly be characterised as "pregnant with thought, and matured by reflection;" but it is, on this account, less suited for the lower than the higher classes of the profession. There is a terseness, a precision, and an aphoristic conciseness in every sentence, that will assuredly cause it to appear dry, or even obscure, to the sciolist, and to the less studious members of the medical world; but which must render the work more valuable to those who look beyond the surface of things, and study with care the mechanism, the functions, and the lesions of the human frame.

The extent of our analysis will, therefore, be proportionate to the value which we set upon the publication; and we cannot pay a greater compliment to the author, or confer a greater benefit to our readers, than by allotting a portion of our Review to the dissemination of Dr. Parry's labours, which will at once evince the estimation in which we hold them, by the reluctance with which we shall part from them.

The work is divided into one thousand and twenty-three aphorisms or passages, of unequal lengths, and apparently without little regard to order or arrangement. This *plan* we humbly conceive to be injudicious, inasmuch as it is very uninviting to the reader; but Dr. Parry, no doubt, trusted to the sterling merit of the *matter* without shewing any very fastidious regard to the *manner* in which it is conveyed.

After some observations on the nature of medical science, our author enters on a consideration of the disordered states of the sanguiferous system, which form the most obvious deviations from health, of any incidental to the animal frame. These consist in some excess or defect in the quantity or velocity of the circulation. The *quality* of the circulating mass is left out of consideration. The *general* velocity of the blood may be natural, while in particular *parts* of the body it may be retarded or accelerated. So also in regard to quantity; the excess or deficiency may be either general or local; that is, there may be excess in one part and deficiency in another, or there may be general excess or deficiency in every part of the system at the same time. *Excessive* quantity is denoted by quick motion of the heart; preternatural fulness of the arterial, capillary, and venous system, and vice versa. The habits of mankind in civilized society tend to produce excessive nutrition, and consequently plethora in the human frame. This state of the sanguiferous system characterizes nine-tenths of the diseases of persons in moderately affluent circumstances. Blood is the pabulum from which, as it circulates through the capillaries or elsewhere, caloric is sensibly evolved, by whatever power that evolution may be effected. An increase of momentum and quantum in the circulation of a part is characterized by pre-ternatural redness and size of that part, and especially if the arteries leading to, and the veins receding from that part be unusually distended. The *quantity* of blood in the whole system remaining the same, a diminution in one part must produce plethora in all, or some part of, the system, and vice versa. The same may be said of the *momentum* of the blood. The coats of arteries possess two powers of motion; the first is mechanical elasticity, the other a vital or muscular power; denominated by our author tonicity. It is something similar to muscular action, but our author denies that the middle coats of arteries are *fibrous*. They have, however, " an inherent capacity of motion," and that is enough. In the larger arteries the mechanical; in the capillaries, the *tonic* power prevails. Our author believes with Haller, that the force of the heart *alone*, in health, is adequate to the task of carrying on the march of the blood through its entire circulation. He concludes, that muscular contraction in the vessels themselves, would just as much impede the ingress of a new quantity of blood, as promote the egress of that already existing in them. To this we do not give implicit assent, but it is not of very great consequence. However it may be explained, it is known, that certain vessels become unusually distended with blood, not only from excessive impulse by the heart, but *without* that impulse, as where shame fills the cutaneous arteries of the face, neck, &c. One of the most important phænomena of the animal frame is, that after vessels have been more or less robbed of their blood, a contrary state succeeds, termed *reaction*, altogether inexplicable, but on the principles of vitality. From our author's observations on the structure and functions of the sanguiferous system, we shall only extract the following passage.

"The arteries and veins being in a state of health always full, and the blood being incompressible, that fluid may be considered as if it formed a continuous solid column all the way onwards from the mitral to the tricuspid valves. Hence, at every systole or contraction of the left ventricle of the heart, the shock acts at the same instant throughout the entire circle." 34.

Our author justly observes, that could we calculate, from the heart's action, respecting the proper quantity of blood in the whole, or any part of the system, great benefit would result; but the pulse is now as it was two thousand years ago—"res fallacissima." Thus, in many diseases, the pulse of the radial artery will be small, while that of many other tangible arteries will be full and strong. Again, the face and head will often be flushed and hot; the pulse of the carotids strong, full, and bounding; while all the extremities are cold and pale, and the pulsation of their arteries small and weak. In gout, and local inflammations without fever, the arteries leading to the diseased parts will throb violently, while other arteries are tranquil or below par. This remark ought to be kept in mind, as it would often lead to the detection of disease. A general, but not universal law of the human constitution is, that excessive morbid determination to two different parts shall not exist in the same person at the same time. This is exemplified not only in gout, but in several other diseases; for he who has a catarrh to-day, may have a fit of the gout to-morrow; then a discharge of blood from the hæmorrhoidal veins, and shortly afterwards, a rupture of the medullary substance of the brain, from sanguineous effusion. This change of determination is a most interesting subject in animal pathology, and has been long caught at in the treatment of diseases, though with not so much success as could be wished. Thus it is usually in vain that we attempt to solicit to the extremities that excessive determination of blood which is called gout, though we employ topical heat, friction, blistering, &c.

Our author is of opinion, that although a suspension or cure of a disease may, perhaps, be occasionally produced by certain violent causes, (as he had seen asthma suspended by a blow on the head) yet, in the common order of conversions, the new affection or determination is, in point of time, either co-existent with the disappearance of the old one, or more usually subsequent to it; so that in reality, we cannot cure a malady by bringing on the gout, but must first cure the malady, and then, in a predisposed constitution, there is a fair chance that gout will supervene. The same principle is applicable to a great number of other occasions of the highest practical importance, as will be hereafter shewn.

*Inflammation and its consequences.*—After enumerating the various characteristics of inflammation, as it affects different tissues, our author remarks that of that termination strictly called resolution, or return of the colour and size of parts to precisely their former state, without any discharge, he never met with a single instance. In all *these* terminations, our author asserts, and we are perfectly of his opinion, that some exhalation from the over-distended vessels takes place, either into the cellular substance of the part, or neighbourhood, or from the cutaneous surface itself externally, or membranes, as the pleura, peritonæum, &c. internally. Of the other terminations we need not speak, but the foregoing remark is to be borne in mind. When constitutional symptoms accompany local inflammation, if blood be drawn in a projecting stream from a vein in the arm, and received into *small vessels with polished internal surfaces,* and suffered to cool slowly, it will exhibit a coat of fibrine, or what is called the inflammatory buff. But if other portions of blood be taken from the same patient at the same blood-letting, into *larger vessels,* or into one with a rough inside, as queen's-ware, though they be first drawn, and having the freest flow, no such crust will be seen. This ought also to be borne in mind. Although this appearance affords a presumption of some local inflammation, it is not absolutely decisive. It occurs during pregnancy—in morbid determinations, as hæmorrhage, &c.; and also at the commencement of synocha, before any local inflammation exists. It also is occasionally seen in the blood of very old persons, and in those of a *nervous* disposition, where there is total exemption at the time from topical inflammation.

Dr. Parry, by a long chain of reasoning, attempts to prove, contrary to the theory of Dr. Wilson, that in all inflammations, there is not only dilatation and increased quantum of blood in the vessels of the part, but that there is an increased *velocity* or momentum of the vital fluid, particularly when to the *local* inflammation is added *general* fever. Of this increased velocity we much doubt, notwithstanding the arguments of our author; nor has he convinced us of the "influence of the systole of the left ventricle on the returning venous blood," because "it frequently happens, that in gouty inflammation of the wrist, blood taken from the cephalic vein is propelled *in jets*, as strong as those from the temporal artery, and precisely synchronous with the pulse in the radial artery in the other arm." 78. We think that this curious phænomenon (which we have witnessed in many instances where there was no gout or local inflammation at all) might be accounted for otherwise than by supposing that the systoles of the heart "extended their projectile force through the capillaries, and nearly back through the entire course of the circulation." 79. Dr. Parry offers some ingenious and sensible observations on what is termed the "*proximate cause*" of a disease, defining it—"that phænomenon, in the body or part, most *immediately preceding* the state which we call disease, without which previous phænomenon, the disease is not known to exist." 91. In this point of view, an increased momentum in the velocity of the blood through a part, and also a dilatation of the vessels of that part, may be considered as the proximate cause of inflammation.

Speaking of the process by which Nature cures an inflammation, our author justly observes, that after increased action, the heart generally falls into a state of unusual quiescence; while, at the same time, the general mass of blood is more or less diminished: (for instance, by the diminution of increment during all inflammatory affections.) It is, therefore, difficult to say, whether, an exhaustion of the heart, in consequence of over exertion, or an abstraction of a quantity of that blood which is so great a stimulus to its action, be, in this case, the chief cause of its quiescence: both probably concur. 94.—On the other hand, if, by undue stimulation, too sudden a restoration of the plethoric state succeeds, and the heart is again excited to excessive action, the local disease, or some vicarious one, is readily produced. This we have so frequently seen bring on relapses after febrile diseases, that we heartily concur with Dr. Parry, when he deplores the mischief that too often results, "when, under the delusive notion of strengthening the patient, he is pampered with full diet, and stimulated with every description of drachms, whether solid or fluid, which the elaboratory of the cook, the vintner, or the apothecary can supply." 96.

In fact, the quantity of blood, suited to the salutary performance of the circulation, is by no means always the same, even in the same person. Thus, a man in the prime of life and health shall habitually have a considerable fulness of the sanguiferous system, yet enjoy an exemption from disease ; " but should the same man, after having been emaciated, suddenly grow full of blood, some violent disease, or succession of diseases, will usually again occur, and reduce the habit to its former state of emaciation."

A multifarious series of this kind will sometimes occur during convalescence, from long and violent diseases ; and, in other cases, where health and strength have succeeded to great extenuation, and have continued for months, and even years, no sooner has the habit, by degrees, recovered its prior fulness, than the same, or a similarly acting disease, shall return, and reduce the patient to another emaciation. This course we see continually exemplified in gout, erysipelas, and various other diseases, which are called constitutional; " and indeed, they are so frequent, says Dr. P. that one cannot help considering the increased action of the heart, and the general circumstances of the disease connected with it, as natural efforts to remove a degree of fulness incompatible with the due performance of the healthy functions of that individual constitution." 97.—Under circumstances of *previous reduction*, from whatever cause, what morbid effects do we not daily see, even from an improper meal—from a strong mental emotion—from watching—wine, &c. which, in health, would never be noticed? Is it not, that in such cases, the heart is more susceptible of the stimuli applied to it?

Dr. Parry next enumerates the various structures of the human body, according to the arrangement of Bichât, and while he admits that each tissue is subject to its own peculiar modifications of disease, *in general*, he yet remarks, that the disease of one tissue will sometimes spread to another; and doubts " whether, in *all* the different textures, there is not *one* component or constituent part which is primarily affected in that morbid change called inflammation." 100.

It has been before remarked, that all terminations of inflammation are attended with more or less extravasation, and the same so commonly occurs in cases of general increased impetus, as in sweating after strong exercise, exposure to heat, palpitation of the heart, fever, &c.; in all of which, a reduction of the increased impetus follows the effusion, that we cannot help conceiving the evacuation to be a process expressly designed for the purpose of mitigating or curing the disease.

Wherever important functions are to be performed, there we find a large supply of blood, by means of arteries terminating in an infinite number of capillaries, and disposing those very parts particularly to congestion or inflammation. But the benevolent author of our existence has made ample provision against these occurrences. Thus these various parts or organs are abundantly furnished with exhalents or secretory vessels from their capillaries. First apparatus, a simple surface, as the skin and mucous membranes. Second, natural cavities; the internal surface of which is lined with similar membrane as the stomach, bowels, bladder, &c. Third, discontinuity of substance, forming virtual, though not often real cavities, into which exhalents open, as the cellular system, ventricles of the brain, medulla oblongata, nerves, duplicatures of the pleura, peritonæum, pericardium, synovial receptacles, &c. Fourth, an excretory duct or ducts, as in the mammæ, liver, kidnies, &c. &c. Most of these organs have a combination of two of these circumstances of structure, so as to acquire a double power of evacuation. Thus the lungs have pleura without, and mucous membrane within. The liver, peritonæum without, and pori biliarii from within. To these may be added, the cellular, parenchymatous, and other substances affording a third emunctory for the superfluous contents of blood-vessels, by means of exhalents and secretory capillaries every where opening into them. To these contrivances there are only two exceptions, the thyroid gland and spleen.

The extravasated fluids resulting from inflammation are various. The simplest form is that of serum effused into the cellular membrane, as in superficial gouty inflammation; it is a true dropsy or œdema of the part. The same occurs as a termination of various inflammations; as of the gums in caries, &c. of the teeth; of the leg, in sciatica; of the joints, in rheumatism; of the lungs, in peripneumony; of the forehead, temples, and eyelids, in erysipelas; in all parts, from external injuries, as blisters, &c. The same kind of effusion is poured out on the surfaces of the serous membranes, as the pleura, pericardium, &c. It is not confined, however, entirely to serous membranes:

"For in certain cases of pulmonary hectic an expectoration often occurs of a dense, hard, globular substance, partly transparent, and partly of a pearly whiteness, which immediately falls to the bottom of the water into which it is thrown, and has much the appearance of thickened albumen." 107.

Dr. P. is of opinion that albumen and fibrine are increased by all inflammatory processes in the constitution. The deposition of fibrine in particular, Dr. P. thinks, may account for various painless, but hard and obstinate swellings, which succeed inflammations, especially about the tendons and joints. Our author believes, and we think with reason, that small parts of the body may be made to unite again, after having been entirely separated, as Dr. Balfour and Mr. Bailey have indeed lately proved.

Another termination of inflammation is the effusion of blood itself. We see this in the bloody spots or streaks in the sputa of bronchitic and pneumonic patients, which, when slight, are always favourable. In pulmonic and hepatic inflammation, however, blood is often *fatally* effused into the air-cells and pori biliarii. Even from the surfaces of serous membranes entire blood is sometimes exhaled, especially about the period of death, both previously and subsequently. That termination of inflammation denominated Pus is not, even to this day, exactly characterised, since it (pus) is hardly ever procured unmixed with other fluids. Dr. Parry is inclined to believe that real pus may be secreted from unbroken surfaces.

"In cases of hemiplegia," says he, "in which blood is effused into the medullary substance of the brain, that fluid may be seen in all the intermediate states from entire blood, through what in appearance exactly resembles pus, to complete absorption." 122.

Dr. P. next takes a review of the various morbid, or rather *salutary* effusions and secretions which result from inflammatory processes, or increased momentum of the blood; previously observing, that "on a great variety of occasions, the effect of inflammation is to increase the natural secretions of the parts affected." But we are of opinion that, "on a great variety of occasions," the *contrary* is the case, and particularly in one of those instances which he has brought forward in corroboration of his opinions, viz. hepatitis. We have had opportunities of seeing this disease on a much larger scale than Dr. Parry; and from very attentive observation, we believe we may say with safety, that, during inflammation of the liver, there is in reality a *paucity* of the biliary secretion. We know, indeed, that discharges of morbid bile sometimes take place during the inflammatory process, and are speedily ejected from their irritating qualities; but such occurrences are not to warrant the conclusions which our author has drawn. Our author is of opinion, and with reason, that although these discharges from inflamed vessels are sometimes fatal (as in hydrocephalus or pneumonia for instance), " the process, in a great majority of cases, is beneficial to the animal frame." 128. Among the fluids effused from inflammation, Dr. P. enumerates that cream-like substance deposited in the cavities of joints, in capsular ligaments, in the sheaths of tendons, &c. which, by the absorption of the thinner parts, becomes what is called chalk-stone, a well-known effect of highly inflammatory gout, and consisting of urat of soda, The same is deposited in the cellular space between the inner and fibrous coats of the larger arteries, becoming true bone, or phosphate of lime, and producing such distressing effects, resulting, according to our author's observations, from an inflammatory affection of the vasa vasorum; and when on the coronary arteries of the heart, predisposing to syncope angens. Nodosity of the joints, Dr. P. supposes to be owing to " an erroneous deposit of common bone."

It has been shewn, that the capillary arteries have a power of contracting to their natural size, after being preternaturally dilated with blood. This power, however, seems to diminish, *cæteris paribus*, in proportion to the violence and duration of the distending cause:

> " Hence," says our author, " one can scarcely avoid considering the state which follows, as what Dr. Cullen would have called collapse, consequent on undue excitement; and Dr. John Brown, indirect debility. But whatever name may be given to this state, the order of facts is not the less true." 135.

Dropsy. One of the simplest and most common terminations of inflammation is extravasation of serum, or one of its constituents; thus the swelling, which often accompanies the cessation of gouty paroxysms, is a true dropsy of the anasarcous kind, following, in free spaces, the direction of gravitation: " where, however, it is considerably extensive, it seems to arise *against gravitation*, relatively to the inflamed part." This phænomenon, Dr. P. thinks, may be explained by supposing that the expulsory power of the exhalents overcomes the force of gravity in a column confined all around. Since we find that in gout œdema continues to extend itself in proportion as the inflammation in the foot subsides, and often exists long after all symptoms of local inflammation are gone, " we cannot help," says Dr. P. " attributing its occurrence, in the latter case, to such a state of momentum of blood still existing in the vessels leading to the part originally inflamed, as produces preternatural evacuation by exhalation." And, on the same principle, the anasarca or œdema, extending to a distance from the original *seat* of inflammation, " originates in a similar condition in the neighbouring arteries and exhalents; both of which appear, from all the phænomena in such cases, to be preternaturally distended with blood." 141. Thus, in œdematous swellings of a lower extremity, following gout in the foot, if we apply a bandage to the foot and ankle, we shall still find an œdematous swelling recur every night *above* the bandage, and often in a greater degree than before the bandage was applied. Hence we may conclude, that *increased momentum* is sufficient to produce anasarca or œdema, without the existence of local inflammation as the source from whence the effusion takes place. Dr. P. illustrates this reasoning by the œdematous swellings of lower extremities following scarlatina, and also the ascites which not unfrequently supervenes, both evidently originating in the high phlogistic diathesis then prevailing in the system.

The fluid effused in what are supposed to be idiopathic ascites, hydrothorax, and anasarca, has all the chemical qualities of common serum; and the identity of the fluid thus effused with that which arises from certain degrees of inflammation in the same membranes, is certainly a strong argument in favour of a state in them approaching to the phlogistic diathesis. 143. This idea is farther strengthened by the thickening and opacity of the peritonæum and pleura, unaccompanied with disease of the liver or other part, which we see in ascites and hydrothorax, and which would justify our inferring a previous topical inflammation in those membranes. This inflammation proceeds in so slow and chronic a manner, with little or no pain or fever, and with only some symptomatic irregularity in the alvine excretions, that the patient is scarcely aware of the existence of important disease till he is alarmed by a preternatural tumefaction of the belly. So, our author has seen hydrothorax slowly arise from a diseased state of the pleura following slight inflammation, accompanied with habitual fever and high-coloured urine, but without the smallest affection whatever of the organs of respiration, till after the lapse of several months, when, at the end of a few days, the patients died, and dissection exhibited copious serous ex travasation, without any pulmonary disease. 145. Anasarca of the lower extremities is also often preceded in them by local pains, which do not go the length of inflammation, and subside as the effusion takes place. In such cases, we cannot reasonably expect to find the secreting membranes to be always in a morbid state; for here, as in acute inflammation, the exhalation is the cure, or at least the effect of the curative process of the excreting parts. Hence the explanation of Bichat's assertion, that in increased secretions, the vascular system supplying the part, is not generally found more full than on other occasions.

The fluid found in the cavities of the brain in hydrocephalus is not serum, or at least contains little of it. It is not coagulable by heat or acids, though a small proportion of albumen is precipitated from it by the voltaic pile. It is worthy of attention, that dropsy is often evidently produced, and when existing aggravated, by many of those circumstances which are known to increase the momentum of the blood, as intemperance gives rise to ascites and anasarca without hepatic disease. From all these circumstances, it is probable, that although serous effusions are usually the consequences either of local inflammation of cellular or serous parts, yet they may occasionally take place from a degree of excessive momentum short of that which would have been necessary to produce either of these two states. As inflammation rarely occurs till some time *after* increased momentum of blood, so extravasation uniformly obeys a similar law, as may be illustrated by the obvious example of sweating, which rarely supervenes till a considerable time after the increased action of the heart from heat, exercise, &c. On other occasions of increased momentum, as in inflammation, effusion is not always proportioned, either to the mere disposition in the capillaries, or to the degree of increased momentum; but is relative to the sum of the two taken together. 148.

"Hence it will be found, that in certain states of the capillary system, even the healthy impetus may be sufficient to cause effusion; while, in other states, very great degrees will not produce the same effect, either at all, or else only through the medium of local inflammation." ib.

This principle, our author thinks, will comprehend all the more essential examples of idiopathic dropsy, as well those styled *active*, as those attributed to debility.

"In this view, the malady in question, and the state of inflammation, throw on each other reciprocal light; since it appears that both have in common the circumstance of a degree of momentum of blood, which is excessive with regard to the whole, or a part of the animal system in general, or of that particular animal, or accidental constitution of the animal, which is the subject of the malady." 149. "If these facts be well founded, general dropsy, like the extravasations in inflammation, is to be considered rather as a salutary effort of the constitution to diminish morbidly increased momentum, than as a primary or actual disease:"

Though in this, as in the former case, the effusion occurring in certain parts, as in the ventricles of the brain, may prove fatal. This principle also illustrates the efficient and final causes why dropsy of various kinds follow venous and glandular obstruction; the arterial blood, thus arrested in its natural course, pouring out its serum through the exhalent extremities of the containing vessels. The phlogistic diathesis, our author thinks, may not be essential to serous effusion, or dropsy; though few dropsies will be found to exist without the appearance of inflammatory crust in the blood, at some stage or other of the disease. We may also conceive that easily yielding state of vessels, approaching to that of death, which, in order to the production of effusion, does not require the coincidence of excessive momentum.*

It ought always, however, to be kept in mind, that in all these cases, the impetus is excessive with regard to the individual constitution, and therefore is speedily followed by the process of effusion. Dr. Parry remarks, that diminished absorption, as a cause of dropsy, is utterly untenable, his observation not having furnished him with a single fact in support of the agency of this cause. We fully agree with him. We have thus analysed the first third part of the volume before us, as a specimen of our labours, and we leave the reader to judge of our success. The analysis of Dr. Parry's work will be continued through the next two Numbers of the Journal.

* Vide Dr. Seeds on Blood-letting, in a subsequent part of this Number.

