# Corrigenda

**Published:** 1816-02

**Authors:** 


38, l. 14, from bottom, *for* little, *read* much

